# Breast Cancer and Bone Mineral Density in a U.S. Cohort of Middle-Aged Women: Associations with Phosphate Toxicity

**DOI:** 10.3390/cancers15205093

**Published:** 2023-10-21

**Authors:** Ronald B. Brown, Philip Bigelow, Joel A. Dubin

**Affiliations:** 1 School of Public Health Sciences, University of Waterloo, Waterloo, ON N2L 3G1, Canada; pbigelow@uwaterloo.ca (P.B.); jdubin@uwaterloo.ca (J.A.D.); 2 Department of Statistics and Actuarial Science, University of Waterloo, Waterloo, ON N2L 3G1, Canada

**Keywords:** breast cancer, bone mineral density, phosphate toxicity, dysregulated phosphate metabolism, tumorigenesis, metastatic breast cancer, osteoblastic skeletal lesions, osteolytic skeletal lesions

## Abstract

**Simple Summary:**

Phosphate toxicity, the accumulation of toxic levels of phosphate in the body, is associated with tumor growth and bone mineral abnormalities. Based on shared associations with phosphate toxicity, the hypothesis of the present study proposes that breast cancer is associated with bone mineral abnormalities in middle-aged women from the U.S. Study of Women’s Health Across the Nation. The results of the present mixed methods–grounded theory study confirmed that women self-reporting breast cancer had a greater magnitude of changes in bone mineral density over time compared with women who remained cancer-free. These findings have implications for phosphate toxicity as a potential cause of bone metastasis in metastatic breast cancer, and future studies should investigate a low-phosphate diet to reduce tumorigenesis and bone mineral abnormalities in breast cancer patients.

**Abstract:**

Breast cancer is associated with phosphate toxicity, the toxic effect from dysregulated phosphate metabolism that can stimulate tumorigenesis. Phosphate toxicity and dysregulated phosphate metabolism are also associated with bone mineral abnormalities, including excessive bone mineral loss and deposition. Based on shared associations with dysregulated phosphate metabolism and phosphate toxicity, a hypothesis proposed in the present mixed methods–grounded theory study posits that middle-aged women with incidence of breast cancer had a greater magnitude of changes in bone mineral density over time compared with women who remained cancer-free. To test this hypothesis, a mixed-effects model was used to analyze the associations of breast cancer incidence with spinal bone mineral density changes in the U.S. Study of Women’s Health Across the Nation. Compared with women in the cohort who remained cancer-free, women who self-reported breast cancer had higher bone mineral density at baseline, but had more rapid losses in bone mineral density during follow-up visits. These findings agree with the hypothesis that a greater magnitude of changes in bone mineral density over time is associated with breast cancer in a cohort of middle-aged women. The findings also have implications for studies investigating dysregulated phosphate metabolism and phosphate toxicity as causative factors of bone metastasis in metastatic breast cancer. Additionally, the authors previously found increased breast cancer risk associated with high dietary phosphate intake in the same cohort of middle-aged women, and more studies should investigate a low-phosphorus diet to reduce bone mineral abnormalities and tumorigenesis in breast cancer patients.

## 1. Introduction

An association of breast cancer with high bone mineral density (BMD) has been reported in the literature [1], but the underlying causative mechanisms of this relationship have not been established. For example, a 2013 meta-analysis of ten prospective studies involving 70,878 postmenopausal women found that high BMD was associated with increased breast cancer risk [2]. Also in 2013, a retrospective study of Israeli women found an association between breast cancer and higher BMD in the lumbar spine, femoral neck, and total hip [3]. A more recent case–control study in 2019 confirmed that breast cancer in Brazilian women was associated with high BMD in the lumbar spine, but not in the femoral neck or total femur [4]. Interestingly, a 2022 case–control study reported that BMD in women with breast cancer was higher compared with those in a control group, even though the breast cancer cases had lower average vitamin D levels, which are normally associated with bone health [1].

Further contributing to the research literature on bone mineral density and breast cancer is the opposite finding of increased osteoporosis risk associated with breast cancer in postmenopausal women, suggesting that breast cancer may share common “biochemical links” with low bone mineral density [5]. However, treatment for breast cancer is also associated with bone loss [6], and treatment effects must be considered in assessing osteoporosis risk associated with breast cancer in women. On the other hand, hormone replacement therapy (HRT) increases BMD, and HRT is also associated with increased risk of breast cancer [7]. These findings suggest that both high and low BMD may be biochemically linked to breast cancer through unknown factors.

Adding to the controversy, other studies have failed to find an association between breast cancer and BMD [8,9,10,11]. Part of this inconsistency in study findings may be explained by the differing intervals of repeated follow-up measures to detect longitudinal changes in bone mineral density related to the incidence of breast cancer [9]. Importantly, healthy bone mineral density levels are neither excessively high nor low, and elevated bone mass has been associated with degenerative bone disease, such as osteoarthritis [12,13]. Furthermore, phosphate toxicity, the pathogenic effect of dysregulated phosphate metabolism in the tissues of the body, is associated with tumorigenesis [14] and negatively impacts bone health [15]. Yet, no studies have investigated phosphate toxicity and dysregulated phosphate metabolism as factors associated with high and low levels of bone mineral density in breast cancer. A brief description of phosphate metabolism and phosphate toxicity follows.

The metabolism of serum inorganic phosphate (Pi) is regulated through endocrine hormones secreted by the bone–kidney–intestine–parathyroid axis [16]. Intestinal absorption of Pi is increased as the kidneys release the bioactive form of vitamin D, 1,25(OH)_2_D_3_, also known as calcitriol. The kidneys reabsorb Pi to maintain normal serum Pi levels and excrete excess Pi in the urine. Fibroblast growth factor 23 (FGF23), released from bones, and parathyroid hormone (PTH) released from the parathyroid glands, help to regulate Pi levels by inhibiting kidney reabsorption of excessive Pi and increasing urinary phosphate excretion.

Phosphate toxicity from excessive accumulation of phosphate in the tissues of the body can accelerate aging, cause bone deformities, and reduce longevity [17]. Importantly, hyperphosphatemia (excessive amounts of Pi in the serum) can lower the serum calcium levels, triggering PTH to resorb bone and release calcium into the serum to restore normal levels of calcium. Dysregulated amounts of serum Pi also increase calcium phosphate levels, increasing ectopic calcification throughout the body, including calcium phosphate deposits of hydroxyapatite in soft tissues and bone [16]. Moreover, high calcium-phosphate product is associated with C-reactive protein [18], and C-reactive protein is associated with bone mineral loss [19].

**Figure 1:**
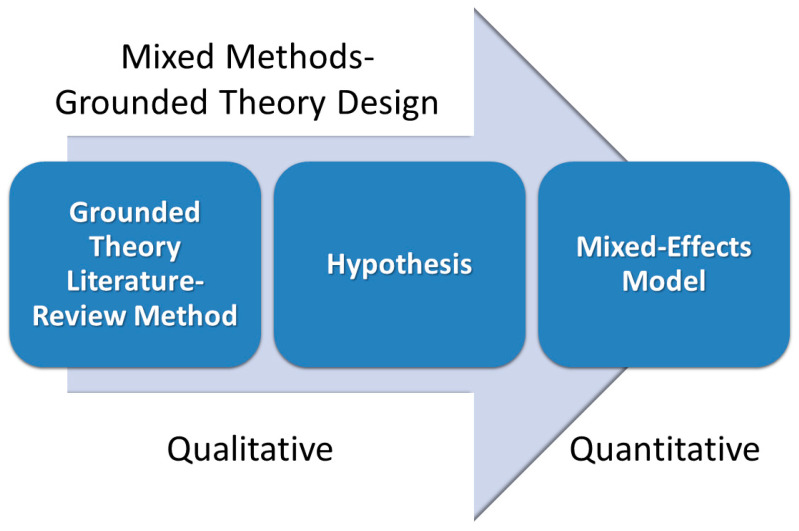
Mixed methods–grounded theory design.

Using a mixed-methods approach to analyze both quantitative and qualitative data [20], the present study investigates the longitudinal changes in bone mineral density associated with breast cancer incidence in the U.S. Study of Women’s Health Across the Nation (SWAN) [21]. The authors previously found a 2.3 relative risk of breast cancer in the SWAN cohort associated with high daily dietary phosphate intake of >1800 mg compared with 800–1000 mg (RR: 2.30, 95% CI: 0.94–5.61, *p* = 0.07) [22]. The present study uses a mixed methods–grounded theory design (MM-GT) to combine qualitative and quantitative data in theory development [23]. This study follows a MM-GT design similar to the three phases described by Shim et al. [24]: a qualitative exploratory and theory development phase, a quantitative confirmatory phase, and a final integration phase. In the present study, the research literature was rigorously and objectively reviewed using a grounded theory literature review method, as described by Wolfswinkel et al. [25], and a hypothesis was generated for quantitative testing using a mixed-effects model (Figure 1). The results of the quantitative analysis were then integrated with the qualitative evidence in the final discussion of this paper.

## 2. Qualitative Analysis—Grounded Theory Literature Review

In the qualitative analysis of the present MM-GT study, research findings on phosphate toxicity, breast cancer, and bone mineral density were selected from all relevant sources for comparative analysis of concepts consisting of pathophysiological relationships and mechanisms. Concepts are the building blocks of qualitative analysis in the present MM-GT study.

*“Concept formation in qualitative research is a systematic process whereby the researcher sets definitions for important concepts that emerge during the research. These definitions help to provide the parameters for the qualitative study”.*

[26]

Concepts were organized into themes and eventually synthesized into a novel qualitative theory explaining the metabolic mechanisms by which phosphate toxicity and dysregulated phosphate metabolism are associated with changes in bone mineral density and tumorigenesis, i.e., the “sensitizing concepts” that guided the development of the MM-GT study [27]. 

### 2.1. Phosphate Toxicity and Tumorigenesis

The following includes a brief summary of the findings cited in a review of phosphate toxicity and tumorigenesis [14]. Elevated levels of Pi within the tumor microenvironment stimulate cell signaling in tumorigenesis [28] and stimulate tumor neovascularization in lung and breast cancer cells [29]. Excess phosphate uptake into the nuclear RNA of cells was shown to stimulate tumor growth, which was delayed when phosphorus uptake was suppressed [30]. Sodium phosphate cotransporters that sequester extracellular Pi are overexpressed in cancer cells of the ovaries, lungs, breasts, and thyroid gland [31,32]. The rate of transport of high Pi concentrations into breast cancer cells through H+-dependent Pi transporters is five times that of sodium phosphate cotransporters [33]. Additionally, a letter published in *Science* as far back as 1946 noted the detection of breast tumors through increased uptake of the radioactive isotope phosphorus-32, compared with lower uptake of the phosphorus isotope by normal breast tissue [34]. A comparison of mouse models also showed that the effects of cachexia in cancer were similar to the effects of phosphate toxicity, with sarcopenia (muscle-wasting), osteoporosis, spinal kyphosis, and organ atrophy [35].

Hyperphosphatemia in patients was associated with chromosome instability and increased proliferation of parathyroid cells [36], and elevated levels of Pi in extracellular tissue were associated with cancer metastasis in a mouse model of breast cancer [37]. High dietary intake of phosphate in the Health Professionals Follow-Up Study was associated with high-grade prostate cancer [38], and another study found that serum phosphate levels were abnormally higher in cancer patients compared with control patients [39]. Experimental animals fed high-phosphorus diets developed lung tumors [40] and skin cancer [41]. Furthermore, tumor cells of the lung and colon in humans contain up to twice the amount of Pi as normal cells [42].

### 2.2. Bone Remodeling and Dysregulated Phosphate Metabolism 

Normal bone metabolism renews bone tissue through a balance of mechanisms that break down and remove worn bone tissue, and replace the discarded tissue with deposits of new bone:

*“Bone remodeling is the process by which bone is renewed to maintain bone strength and mineral homeostasis. Remodeling involves continuous removal of discrete packets of old bone, replacement of these packets with newly synthesized proteinaceous matrix, and subsequent mineralization of the matrix to form new bone. The remodeling process resorbs old bone and forms new bone to prevent accumulation of bone microdamage”.*

[43]

If bone remodeling mechanisms that normally build up and break down bone become unbalanced, metabolic bone disorders may occur, such as osteoporosis, in which “bone resorption outpaces bone formation” [44]. Of relevance, mineral and bone disorder is associated with chronic kidney disease (CKD-MBD), in which serum Pi homeostasis is often dysregulated [45,46]. Additionally, “studies have shown that patients with chronic renal failure (CRF) are more likely to suffer from breast cancer and other malignant tumors” [47]. Furthermore, dysregulated phosphate and phosphate toxicity potentially mediate an association of mineral bone disorder with breast cancer by causing excessive release of PTH in hyperparathyroidism (known as secondary hyperparathyroidism).

*“PTH can produce catabolic or anabolic effect(s) on bone metabolism depending on the level of the hormone, periodicity, and duration of exposure”.*

[48]

Loss of healthy bone in cancer is found in combination with increases in abnormal bone deposits, or osteoblastic skeletal lesions [49,50]. Abnormal calcification of bone is seen in metastasis of the breast, prostate, and other cancers [51]. Bone deposits are also associated with osteosclerosis, a hardening in which excess minerals are abnormally deposited into the bone matrix [52]. The main causes of osteosclerosis include secondary hyperparathyroidism [53], which is commonly associated with hyperphosphatemia in renal insufficiency [54]. “It has already been established that in end-stage renal disease, hyperphosphatemia causes soft tissue calcification,” and dysregulated phosphate metabolism may be responsible for the observed associations of calcification in normal populations [55]. Additionally, ectopic calcification from calcium phosphate deposits in the form of microcalcifications of the breast has been associated with increased risk of breast cancer [56].

Low vitamin D levels associated with dysregulated phosphate metabolism are common in CKD [57], and breast cancer risk is inversely associated with levels of vitamin D [58]. Breast cancer metastasis is also autonomously promoted by vitamin D deficiency [59]. Furthermore, evidence suggests that increased breast cancer risk is associated with high levels of FGF23 [60] and PTH [61], which are also associated with dysregulated phosphate metabolism.

### 2.3. Metastatic Breast Cancer 

Metastatic breast cancer, stage IV breast cancer that has spread to other organs, is the most advanced form of breast cancer affecting approximately 30% of women with the disease, and is “generally incurable” [62]. Bone is the most common site of metastases in metastatic breast cancer [63]. Importantly, both abnormal bone deposition and bone loss (osteolytic skeletal lesions) appear early in metastatic breast cancer, but breast cancer metastases mostly cause bone loss:

*“Metastases leading to overall bone loss are classified as osteolytic. Those leading to excess bone deposition are considered osteoblastic. However, both bone degradation and deposition likely occur early in the metastatic process. The majority of breast cancer metastases ultimately cause bone loss”.*

[51]

Although breast cancer bone metastases are predominantly osteolytic, 15–20% of breast cancer bone metastases cases “have a predominant osteoblastic component” [64]. Excessive bone deposition in early osteoblastic metastases may account for the increased risk of breast cancer associated with higher BMD. Furthermore, Ramirez and Fielder noted that a “high local phosphate concentration during osteolysis” is observed in breast cancer and bone metastases, which requires further investigation [65]. These findings provide plausible mechanisms by which dysregulated phosphate metabolism and phosphate toxicity are associated with BMD changes in breast cancer.

### 2.4. Hypothesis 

**Figure 2:**
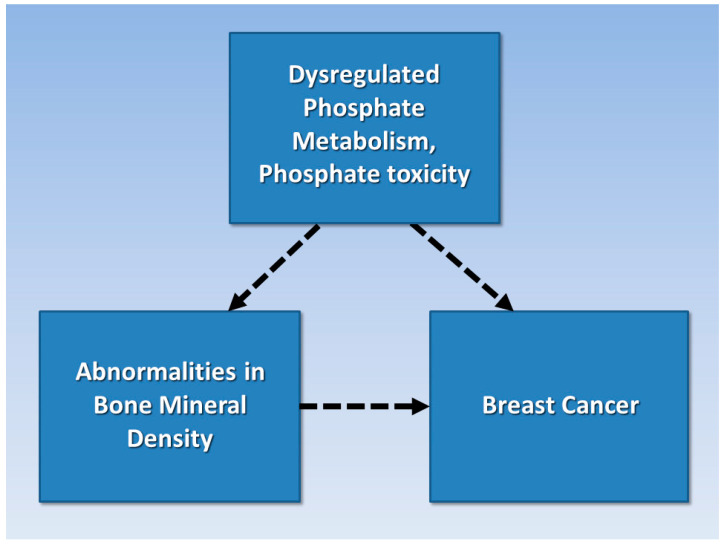
Abnormalities in bone mineral density are transitively associated with breast cancer through shared associations (dashed arrows) with dysregulated phosphate metabolism and phosphate toxicity.

A synthesis of concepts from the previously reviewed literature explains how abnormal bone mineralization and tumorigenesis share associations with dysregulated phosphate metabolism and phosphate toxicity. The rationale used to inform the hypothesis of the present study is based on transitive inference—“the process of inferring the relation between two items based on their shared relation with a third item” [66]. For example, Figure 2 proposes that abnormalities in BMD are transitively associated with breast cancer (dashed arrow) through shared associations with dysregulated phosphate metabolism and phosphate toxicity. Therefore, this study’s hypothesis is that women in the SWAN cohort who self-reported breast cancer incidence during follow-up visits had a greater magnitude of changes in bone mineral density over time compared with women who remained cancer-free.

## 3. Quantitative Analysis—Mixed-Effects Model

The quantitative analysis in the present MM-GT study uses frequent repeated measures to investigate the association of self-reported breast cancer with longitudinal changes in bone mineral density based on a secondary analysis of follow-up data from the SWAN study [21]. The SWAN dataset is a multi-ethnic, multi-site longitudinal sample of middle-aged American women consisting of baseline interviews and examinations of biological, physical, psychological, and social factors, with ten annual follow-up visits [67]. SWAN is co-sponsored by the National Institute of Nursing Research, the National Institute on Aging, the National Institutes of Health-Office of Research on Women’s Health, and the National Center for Complementary and Alternative Medicine. The SWAN dataset and demographic information are freely available to the public online (See Data Availability Statement).

Between 1996 and 1997, 3302 women aged 42–53 years who were free of breast cancer were enrolled in the SWAN cohort [68]. Participants identified themselves as African American (28%), Caucasian (46%), Chinese (8%), Hispanic (9%), or Japanese (9%). In annual follow-up interviews, the participants were asked to self-report any diagnoses or treatments for breast cancer they had received since their last visit. Within the cohort, 2335 women were also enrolled at baseline to receive dual-energy X-ray absorptiometry (DEXA) bone mineral scans of the lumbar spine and femoral neck during follow-up visits [69]. The values in the dataset for bone mineral scans are in grams/cm^2^ for absolute bone mineral density with cross-calibration applied at each visit number.

The present study examined longitudinal data from the SWAN cohort totaling 151 self-reports of cancer incidence in at least one breast and over 17,000 DEXA scans of the lumbar spine (Supplementary S1). The BMD values of the lumbar spine in grams/cm^2^ are listed in the data set as variable SPBMDT. Analysis was performed by fitting a linear mixed-effects regression model to the data using the PROC MIXED statistical analysis procedure in SAS, release 9.04.01M3P06242015. Fixed effects in a mixed-effects model are the constant or fixed relationships assumed between independent and dependent variables, so that “only the dependent variable changes in response to the levels of independent variables” [70]. The fixed effects in the model in the present study quantify the association between spinal BMD in g/cm^2^ (the response variable, SPBMDT) in women self-reporting breast cancer incidence vs. women remaining cancer-free (the main independent variable of interest). In addition to fixed-effect responses in groups, the model’s random effects include the analysis of BMD values from individual participants, which adds more detailed response information to the model. Importantly, random effects are specifically related to some unknown or latent variable in individuals, and “by including random-effects in the model, it is possible for researchers to account for multiple sources of variation” [71].

The general formula for the linear regression mixed-effects model used in the present study is based on Hedeker and Gibbons [72]:

y_*ij*_ = *β*_0_ + *β*_1_*t_ij_* + *β*_2_*x_j_* + *β*_3_(*t_ij_* × *x_j_*) + *υ*_1*i*_*t_ij_* + *ԑ_ij_* (1)

where y*_ij_* denotes the *i*th individual’s continuous BMD values (the dependent variable) at the *j*th repeated measurement;

*β*_0_ is the y-intercept between individuals;

*β*_1_*t_ij_* is the time or trend effect between individuals denoted by the *j*th individual annual visit = 0, 1, 2, 3, 4, 5, 6, 7, 8, 9;

*β*_2_*x_j_* is the *i*th individual’s self-reported breast cancer status (the independent variable) = 1 if yes for breast cancer, 0 otherwise;

*β*_3_*(t_ij_ × x_j_)* is the interaction of *β*_1_*t_ij_* and *β*_2_*x_j_*, the effect of time on the independent variable;

*υ*_0*i*_ is the random y-intercept within individuals;

*υ*_1*i*_*t_ij_* is the random trend effect within individuals;

*ԑ_ij_ is* the residual error within individuals.

### Quantitative Model Selection

The principle of parsimony in statistics “states that a model should be as simple as possible”, whereas overfitting a model with too many parameters “risks identifying spurious factors as important” [73]. The model that best fit the SWAN data in the present study was selected using the Akaike Information Criterion (AIC) [74]. The best-fitting model often has the lowest AIC score, which explains the greatest amount of variation, based on maximum likelihood estimates, and has the fewest independent variables. Maximum likelihood estimation fits a distribution curve to data so that the likelihood that data falls under the distribution curve is maximized [75]. During model selection, a mixed-effects model is scored and compared in a stepwise recursive procedure, adding variables from the general formula of the model one at a time. Moreover, the statistical significance of the variables is another important factor to consider in model selection, and interactive variables, the product of two or more independent variables [76], are also fitted. The specific interactive variable in the present study, the product between time and self-reported breast cancer status, was added to investigate if there were different trajectories of the BMD response over time for women who self-reported breast cancer versus women who did not.

Missing data handled by the present study’s mixed-effects model were assumed missing at random (MAR), meaning that the factors represented by the missing data were unlikely to have contributed to the cause of the data’s absence [77]. Furthermore, maximum likelihood in mixed-effects models has the advantage of forming unbiased estimates with minimal standard error that can consider the uncertainty of missing data, without the need for data imputation [78].

## 4. Results

The hypothesis in the present MM-GT study was tested by analyzing longitudinal data from the SWAN cohort of middle-aged women. A mixed-effects linear regression model was used to examine bone spinal mineral density changes in women who self-reported breast cancer compared with women who remained cancer-free. A stepwise recursive procedure was used to fit the mixed-effects model to the SWAN data (Supplementary S2). Table 1 shows that the fit statistics of the final selected model included the AIC and AICC (corrected for smaller samples) of −60,138.1 and the BIC (Bayesian Information Criterion) of −60,089.8.

The notated formula for the final selected mixed-effects model is:

y*_ij_* = *β*_0_ + *β*_1_*indiv_visit_ij_* + *β*_2_*brstcan_i_* + *β*_3_(*indiv_visit_ij_* × *brstcan_i_*) + *υ*_0*i*_ + *υ*_1*i*_*indiv_visit_ij_* + *ԑ_ij_* (2)

Table 2 lists estimates for the final selected model’s y-intercept, self-reported breast cancer (BRSTCAN), individual visit number (INDIV_VISIT), and the interaction of breast cancer with individual visit (INDIV_VISIT × BRSTCAN). All estimates were statistically significant at *p* < 0.05. Of note, the stepwise recursive procedure (Supplementary S2) shows that the *p*-value of BRSTCAN decreased from 0.8098 to 0.0042 when the interaction of breast cancer with individual visit was added to the final model, indicating a statistically significant longitudinal effect of breast cancer incidence over ten visits.

The final selected mixed-effects model with estimated coefficients from Table 2 is:

ŷ*_ij_* = 1.0837 – 0.00937 *indiv_visit_ij_* + 0.02130 *brstcan_i_* – 0.00411 (*indiv_visit_ij_* × *brstcan_i_*) (3)

**Figure 3:**
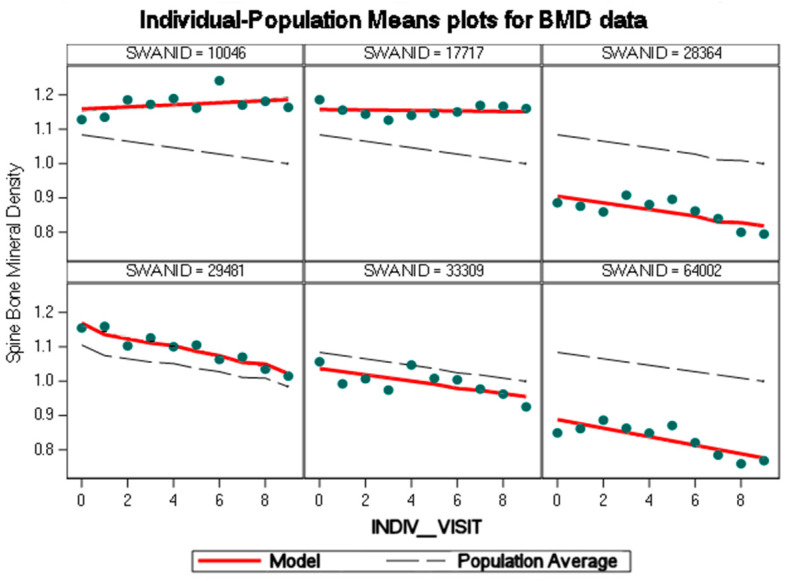
Panel of regression line fit to data (green dots representing BMD per visit).

The panel below (Figure 3) contains BMD values of randomly selected women who were analyzed with the linear mixed-effects model; three women in the upper row who remained cancer-free and three women in the lower row who reported breast cancer. The panel shows that the model fit the regression lines to data exceedingly well, even when data diverged from the population average, implying a small residual variance, ε, in the general formula for the linear mixed-effects model.

**Figure 4:**
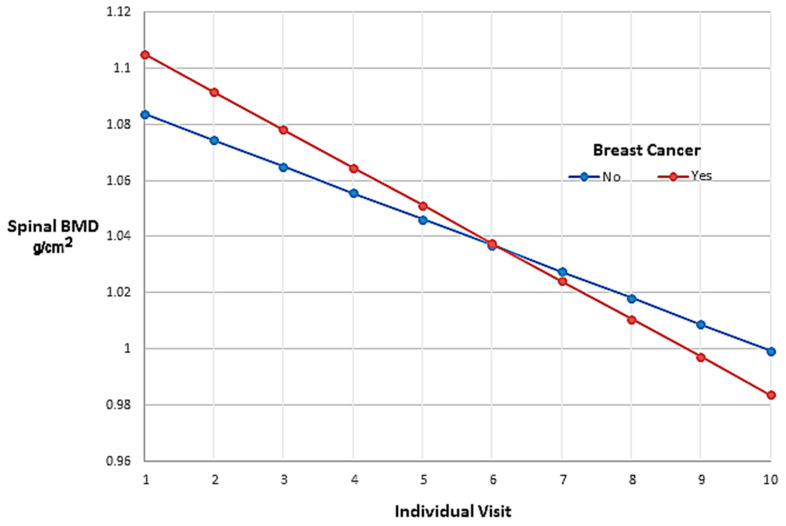
Mixed-effects model regression estimates.

The mixed-effects model’s estimates of spinal BMD values for women in the SWAN cohort throughout 10 individual visits are shown in g/cm^2^ in Table 3. Note that labeling the first visit as 0 begins the model’s estimated BMD of women free from breast cancer at 1.0837 g/cm^2^, the value of the y-intercept. Differences in BMD between the groups showed that BMD in the breast-cancer group was 0.0213 g/cm^2^ higher than that in the other women at the first visit. The rate of BMD decline per visit for each group was 0.01348 g/cm^2^ in the breast-cancer group, which was 0.00411 g/cm^2^ greater than the rate of BMD decline per visit of 0.00937 g/cm^2^ in the cancer-free women. And yet, even with a higher rate of decline in the breast cancer group, the mean BMD in both groups averaged over ten years was almost identical. The spinal BMD values are graphed as linear regression lines in Figure 4.

The graph of regression lines in Figure 4 shows the longitudinal changes in BMD values of women who self-reported incident breast cancer during annual visits compared with women who remained free of breast cancer. The fixed effect of the model shows that, on average, the values for spinal BMD declined over time for all women in the cohort. However, women who reported breast cancer had higher BMD at baseline, which decreased throughout the follow-up periods at a faster rate (steeper declining slope) than that in women without breast cancer. By the end of the study, women who reported breast cancer had crossed over to lower levels of BMD compared with women without breast cancer.

## 5. Discussion

The fixed effect of the mixed-effect model in the present study shows that, on average, all women of the SWAN cohort lost BMD over ten annual visits. However, the random effects of the model showed that women who reported breast cancer during follow-up visits had higher BMD at baseline than women who remained free of breast cancer. This is consistent with other research findings associating high BMD with the risk of breast cancer [1,2,3,4]. Furthermore, women who reported breast cancer lost BMD at a faster rate throughout the follow-up periods, eventually descending to lower BMD levels than those of women free of breast cancer. To the best of the authors’ knowledge, this is the first study of middle-aged women to show a crossover effect from high to low BMD in longitudinal data of breast cancer incidence compared with controls. And yet, the mean BMDs over ten annual visits for each group of cases and controls were almost identical, at 1.04434 g/cm^2^ and 1.04154 g/cm^2^, respectively, a difference of only 0.0028 g/cm^2^. This small difference highlights the advantage of including random effects in the linear regression model to reveal otherwise hidden rate differences in BMD decline between the two groups.

A higher BMD at baseline suggests that middle-aged women who reported breast cancer during the study had progressed through an earlier stage of increased BMD deposition in the years before enrollment in the SWAN cohort. The cohort data do not show the maximum BMD levels attained by these women before enrollment, nor do they show when the incidence of excessive mineralization may have occurred in these women, perhaps coinciding with increasing effects of phosphate toxicity associated with declining renal function. Renal function tends to decrease with advancing age, which is “a normal biological phenomenon linked to cellular and organ senescence” [79], and renal function “seems to diminish with menopause” [80].

The model also shows that BMD in women who self-reported incidence of breast cancer over 10 years was already in decline from the beginning of the annual visits. Furthermore, this finding rules out the effect of cancer treatment on bone loss in women before breast cancer incidence was reported. Additionally, the decline in BMD during follow-up visits rules out the effect of HRT that increases BMD while increasing cancer risk. However, although women were not taking hormones in the three months prior to enrollment in the cohort [67], HRT cannot be ruled out as a factor contributing to increased BMD and increased cancer risk before enrollment in women reporting breast cancer in follow-up visits.

The longitudinal data used in the present model helped to mitigate study design issues and divergent findings in previous studies of BMD in breast cancer. Perhaps the strongest evidence associating the model findings with dysregulated phosphate and phosphate toxicity is that in the recent 2022 case–control study showing that women with breast cancer had higher BMD, despite having low vitamin D levels [1]. Higher vitamin D levels are normally associated with healthy BMD, and lower vitamin D levels are associated with dysregulated phosphate metabolism as the kidneys reduce calcitriol levels to reduce intestinal phosphate absorption. This evidence supports the abnormal nature of elevated BMD associated with dysregulated phosphate metabolism. 

**Figure 5:**
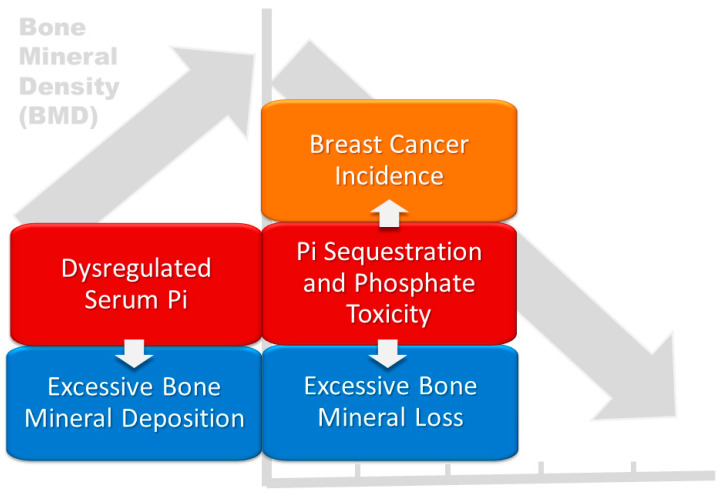
Breast cancer and longitudinal changes in BMD potentially associated with dysregulated serum Pi and phosphate toxicity in the SWAN cohort.

The integration of the foregoing qualitative and quantitative evidence in the MM-GT study supports the findings that a greater magnitude of changes in BMD over time are associated with breast cancer incidence in the SWAN cohort. Furthermore, this association shares associations with phosphate toxicity and dysregulated Pi sequestered in the tumor microenvironment that stimulates breast cancer incidence [14]. Overall, the findings of the present study have implications for bone metastasis in metastatic breast cancer involving dysregulated phosphate metabolism and phosphate toxicity, and more studies are needed in this area. Figure 5 integrates BMD changes and breast cancer in the SWAN cohort, potentially associated with dysregulated serum Pi, and phosphate toxicity.

The limitations of this study include the lack of additional biomarkers linking breast cancer, bone mineral density, and dysregulated phosphate metabolism. For example, future studies should include vitamin D levels and the levels of other endocrine hormones that regulate Pi metabolism, as well as estimated glomerular filtration rates related to renal regulation of phosphate metabolism. Also, the women in the cohort self-reporting cancer diagnoses or treatment may be subject to information bias due to participant errors in recall. Additionally, the associations described in this study are not clinical proof of causation, and more research is needed to confirm the proposed pathophysiological mechanisms relating breast cancer and bone mineral density. Nevertheless, the results of the present MM-GT study may lead to future clinical investigations of dysregulated phosphate metabolism and phosphate toxicity as causes of bone metastasis in incurable metastatic breast cancer. Importantly, “unraveling the biology that governs the interplay between breast neoplastic cells and bone tissue would provide means for the development of new therapeutic agents” [64], and also low-phosphate dietary interventions. 

The findings of this study can inform the development of clinical applications aiming to prevent or reverse the promotion and progression (metastasis) of breast cancer through nutritional interventions that lower patients’ intake of dietary phosphate. Furthermore, the restoration and maintenance of normal serum phosphate levels in patients can be assisted by pharmacotherapies, such as phosphate binders, that reduce the intestinal absorption of dietary phosphorus [81]. The limitations of these clinical applications include the need for trained personnel to instruct, monitor, and guide patients to follow dietary interventions. Patient adherence is also poor for the oral administration of phosphate binders [82], and these medications can be expensive. Fortunately, low-phosphate diets that are safe and effective are already in use for patients with CKD [83]. Applying an interdisciplinary approach, renal dietitians trained to guide CKD patients to adhere to low-phosphate diets could be recruited in feasibility studies to test the hypothesis that a daily low-phosphate diet (800–1000 mg [22]) will help to reduce abnormal bone mineral changes and tumor size in breast cancer patients. Clinical results could be monitored through medical imaging of affected bone and breast tissue within the tumor microenvironment.

## 6. Conclusions

In the present MM-GT study, a grounded theory–literature review method was used to synthesize findings in the research literature, leading to a hypothesis positing that a greater magnitude of changes in BMD over time are associated with breast cancer in middle-aged women. A mixed-effects linear regression model based on the SWAN cohort confirmed that longitudinal BMD changes were higher in women self-reporting breast cancer, but declined at a faster pace than BMD changes in women without breast cancer. Future clinical studies are needed to further investigate the causative role of dysregulated phosphate and phosphate toxicity in BMD abnormalities and bone metastasis in metastatic breast cancer. Furthermore, the authors previously found that high dietary phosphate intake was associated with increased breast cancer risk in the SWAN cohort, and a low-phosphate dietary intervention should be tested to decrease abnormal bone mineral density changes and tumorigenesis in breast cancer patients. Future studies should also monitor the endocrine hormonal levels that regulate phosphate metabolism in breast cancer patients, as well as bio-indicators of decreasing renal function. 

## Figures and Tables

**Table 1:** Fit statistics.

−2 Log Likelihood	−60,154.1
AIC (Smaller is Better)	−60,138.1
AICC (Smaller is Better)	−60,138.1
BIC (Smaller is Better)	−60,089.8

**Table 2:** Model estimates.

Effect	Breast Cancer	Estimate	Std Error	DF	t Value	Pr > |t|
Intercept		1.0837	0.003106	2212	348.88	<0.0001
INDIV_VISIT		−0.00937	0.000201	2121	−46.58	<0.0001
BRSTCAN	Yes	0.02130	0.007439	13E3	2.86	0.0042
BRSTCAN	No [Ref]	0				
INDIV_VISIT × BRSTCAN	Yes	−0.00411	0.001302	13E3	−3.15	0.0016
INDIV_VISIT × BRSTCAN	No [Ref]	0				

**Table 3:** Model estimates of spinal BMD, g/cm^2^.

Visit	Breast Cancer Yes	Breast Cancer No	Difference
1	1.105	1.0837	0.0213
2	1.09152	1.07433	0.01719
3	1.07804	1.06496	0.01308
4	1.06456	1.05559	0.00897
5	1.05108	1.04622	0.00486
6	1.0376	1.03685	0.00075
7	1.02412	1.02748	−0.00336
8	1.01064	1.01811	−0.007046
9	0.99716	1.00874	−0.01158
10	0.98368	0.99937	−0.01569
Mean	1.04434	1.04154	0.0028

## Data Availability

SWAN: Study of Women’s Health Across the Nation. Available online: https://www.swanstudy.org/ (accessed on 2 July 2023).

## Supplementary Materials

The following supporting information can be downloaded at https://www.mdpi.com/article/10.3390/cancers15205093/s1, Supplementary S1: SWAN Data; Supplementary S2: Stepwise recursive procedure to fit model to SWAN data.
