# Supplementary material for: Breast Cancer and Bone Mineral Density in a U.S. Cohort of Middle-Aged Women: Associations with Phosphate Toxicity
Source: Cancers (Basel). 2023 Oct 21;15(20):5093. doi: 10.3390/cancers15205093 (PMC10604967; doi:10.3390/cancers15205093)
Supplement: Supplementary file 1 [file cancers-15-05093-s001.zip › cancers-2642423-supplementary.pdf]

Supplementary S1: SWAN Data

Data in the present MM-GT study include DEXA bone mineral density scans of the spine and self-reported incidents of breast cancer in SWAN participants over 10 annual visits. Out of the original enrollment of 3302 women, the frequency table below shows that 3087 attended the first visit, dropping to 2110 in the last visit.

Individual Visit		
**INDIV_VISIT**	**Frequency**	**Cumulative Frequency**
**0**	3087	3087
**1**	2785	5872
**2**	2727	8599
**3**	2675	11274
**4**	2635	13909
**5**	2386	16295
**6**	2278	18573
**7**	2218	20791
**8**	2180	22971
**9**	2110	25081

Participants self-reported 151 incidents of cancer in at least one breast during annual visits.

Breast Cancer RUN		
**BRSTCAN**	**Frequency**	**Cumulative Frequency**
**No [Ref]**	24927	24927
**Yes**	151	25078

Participants received 2105 DEXA bone mineral density scans of the spine (SPBMDT) in visit 0, and this number dropped to 1539 scans by visit 9.

INDIV_VISIT	SPBMDT_N
0	2105
1	1956
2	1860
3	1780
4	1763
5	1713
6	1657
7	1385
8	1552
9	1539
Total	17310

Supplementary S2: Stepwise Recursive Procedure to Fit Model to SWAN Data

Table S1

Model A: Random Intercept model.

$Y_{ij} = \beta_0 + \upsilon_{0i} + \varepsilon_{ij}$

Fit Statistics	
**-2 Log Likelihood**	-47921.9
**AIC (Smaller is Better)**	-47915.9
**AICC (Smaller is Better)**	-47915.9
**BIC (Smaller is Better)**	-47897.8

Solution for Fixed Effects					
**Effect**	**Estimate**	**Standard Error**	**DF**	**t Value**	**Pr > |t|**
**Intercept**	1.0472	0.003189	2212	328.42	<.0001

$\hat{y}_{ij} = 1.0472$

Table S2

Model B1: Fixed time trend model with Random intercept.

$Y_{ij} = \beta_0 + \beta_1 indiv\_visit_{ij} + \upsilon_{0i} + \varepsilon_{ij}$

Fit Statistics	
**-2 Log Likelihood**	-55250.4
**AIC (Smaller is Better)**	-55242.4
**AICC (Smaller is Better)**	-55242.4
**BIC (Smaller is Better)**	-55218.3

Solution for Fixed Effects					
**Effect**	**Estimate**	**Standard Error**	**DF**	**t Value**	**Pr > |t|**
**Intercept**	1.0841	0.003187	2212	340.16	<.0001
**INDIV_VISIT**	-0.00964	0.000099	15E3	-97.01	<.0001

$\hat{y}_{ij} = 1.0841 - 0.00964 \textit{indiv\_visit}_{ij}$

Table S3

<u>Model B2</u>: Random intercept & trend model.

$Y_{ij} = \beta_0 + \beta_1 \textit{indiv\_visit}_{ij} + \upsilon_{0i} + \upsilon_{1i} \textit{indiv\_visit}_{ij} + \varepsilon_{ij}$

Fit Statistics	
**-2 Log Likelihood**	-60144.1
**AIC (Smaller is Better)**	-60132.1
**AICC (Smaller is Better)**	-60132.1
**BIC (Smaller is Better)**	-60095.9

Solution for Fixed Effects					
**Effect**	**Estimate**	**Standard Error**	**DF**	**t Value**	**Pr > |t|**
**Intercept**	1.0838	0.003106	2212	348.91	<.0001
**INDIV_VISIT**	-0.00939	0.000201	2121	-46.71	<.0001

$\hat{y}_{ij} = 1.0838 - 0.00939 \textit{indiv\_visit}_{ij}$

Table S4

Model C: Effect of BRSTCAN on intercept and trend.

$У_{ij} = \beta_0 + \beta_1 indiv\_visit_{ij} + \beta_2 brstcan_i + \upsilon_{0i} + \upsilon_{1i} indiv\_visit_{ij} + \varepsilon_{ij}$

Fit Statistics	
**-2 Log Likelihood**	-60144.2
**AIC (Smaller is Better)**	-60130.2
**AICC (Smaller is Better)**	-60130.2
**BIC (Smaller is Better)**	-60087.9

Solution for Fixed Effects						
**Effect**	**Breast Cancer RUN**	**Estimate**	**Standard Error**	**DF**	**t Value**	**Pr > |t|**
**Intercept**		1.0838	0.003106	2212	348.91	<.0001
**INDIV_VISIT**		-0.00939	0.000201	2121	-46.71	<.0001
**BRSTCAN**	Yes	0.000881	0.003661	13E3	0.24	0.8098
**BRSTCAN**	No [Ref]	0	.	.	.	.

$\hat{y}_{ij} = 1.0838 – 0.00939 indiv\_visit_{ij} + 0.000881 brstcan_{i}$

AICC is smaller than model B2. BRSTCAN is not significant.

Table S5

Model D: Effect of BRSTCAN*INDIV_VISIT on intercept and trend.

$У_{ij} = \beta_0 + \beta_1 indiv\_visit_{ij} + \beta_2 brstcan_i + \beta_3(indiv\_visit_{ij} \times brstcan_i) + \upsilon_{0i} + \upsilon_{1i} indiv\_visit_{ij} + \upsilon_{ij}$

Fit Statistics	
**-2 Log Likelihood**	-60154.1
**AIC (Smaller is Better)**	-60138.1
**AICC (Smaller is Better)**	-60138.1
**BIC (Smaller is Better)**	-60089.8

Solution for Fixed Effects						
**Effect**	**Breast Cancer RUN**	**Estimate**	**Standard Error**	**DF**	**t Value**	**Pr > |t|**
**Intercept**		1.0837	0.003106	2212	348.88	<.0001
**INDIV_VISIT**		-0.00937	0.000201	2121	-46.58	<.0001
**BRSTCAN**	Yes	0.02130	0.007439	13E3	2.86	0.0042
**BRSTCAN**	No [Ref]	0	.	.	.	.
**INDIV_VISIT×BRSTCAN**	Yes	-0.00411	0.001302	13E3	-3.15	0.0016
**INDIV_VISIT×BRSTCAN**	No [Ref]	0	.	.	.	.

$\hat{y}_{ij} = 1.0837 - 0.00937\,indiv\_visit_{ij} + 0.02130\,brstcan_i - 0.00411(indiv\_visit_{ij} \times brstcan_i)$.

BRSTCAN is significant at 0.0042, and the interaction INDIV_VISIT×BRSTCAN is significant at 0.0016.
